# Body Fat Distribution and Associated Risk of Cardiovascular Disease in Adults With Cerebral Palsy

**DOI:** 10.3389/fneur.2021.733294

**Published:** 2021-12-08

**Authors:** Hyun Iee Shin, Se Hee Jung

**Affiliations:** ^1^Department of Rehabilitation Medicine, Chung Ang University Hospital, Seoul, South Korea; ^2^Department of Rehabilitation Medicine, Seoul National University Boramae Medical Center, Seoul, South Korea; ^3^Department of Rehabilitation Medicine, Seoul National University College of Medicine, Seoul, South Korea

**Keywords:** cerebral palsy, cardiovascular risk, framingham risk score, fat distribution, android fat distribution, adults

## Abstract

**Objective:** Fat distribution has increasingly been acknowledged as a more significant health parameter than general obesity, in terms of the risk of cardiovascular disease (CVD). We aimed to investigate the regional fat distribution pattern and general body fat characteristics of adults with cerebral palsy (CP), and we explored the risk of CVD in this population.

**Methods:** People aged ≥20 years who were diagnosed with CP were recruited between February 2014 and November 2014. The subjects underwent a structured interview, laboratory studies, and physical examination. The amount and distribution of fat were determined directly by dual-energy X-ray absorptiometry. Laboratory analysis was performed to measure total cholesterol and triglyceride, high-density lipoprotein (HDL), low-density lipoprotein, and fasting plasma glucose levels. The Framingham risk score (FRS) was used to present the 10-year risk for having CVD, and predictors such as sex, age, total cholesterol, HDL, systolic blood pressure, treatment for hypertension, and smoking status were used to calculate the FRS.

**Results:** Ninety-nine adults (58 men, mean age 41.77 ± 8.95 years) with CP were included. The participants consisted of all five levels of the Gross Motor Function Classification System. The mean body mass index (BMI) was 22.52 ± 4.58 kg/m^2^. According to BMI criteria, 54.9% were overweight and 27.3% were obese. The fat mass index criteria revealed 10.1% excess fat and 7.6% obesity. In univariable regression analysis, age, the timing of physical function deterioration, and android fat percentage were associated with the FRS (*p* <0.001, *p* <0.001, and *p* = 0.007, respectively). In multiple regression analysis, the FRS was associated with age and android fat percentage, based on the following formula:

$$\begin{array}{l} {}^{“}\text{FRS=}-\text{18}.\text{549 + 0}.\text{410 }*\text{ Age + 0}.\text{577 }*\text{ Android percent fat }\left(\%\right)\;{\left({\text{R}}^{2}\text{=0}.\text{528}\right)}^{\prime \prime }\\ \;\left(p<0.001\right). \end{array}$$

**Conclusions:** Body fat distribution in the android area is significantly associated with future CVD risk in adults with CP.

## Introduction

When people with cerebral palsy (CP) mature into adulthood, they frequently face various secondary conditions. Among the major challenges of this population, lack of physical activity, decreased physical fitness, and a sedentary lifestyle are often reported in adulthood (1, 2). There is a medical concern that these factors may increase the risk of cardiovascular disease (CVD) in the CP population (1–4). Several previous studies have shown that CVD-related mortality is higher in people with CP than in the general population (1, 3, 5).

Physical inactivity in people with CP may increase the risk of obesity. At the same time, they have an increased risk of dysphagia and other gastrointestinal problems, which may lead to nutritional deficiency (6, 7), while spasticity can lead to increased energy consumption (8–10). The reported prevalence of actual obesity in the adult CP population has varied across studies (2, 4).

Recently, fat distribution has been proposed to be more closely associated with CVD risk than with the general measures of obesity, such as total fat mass or body fat percentage (11, 12). Android fat distribution, which refers to the central distribution of body fat, is an important risk factor for future cardiovascular events, independent of overall fat volume (13). More specifically, adults with CP are exposed to secondary musculoskeletal changes, including loss of muscle mass, muscle shortening, joint contractures, and deformity (14). Deficits in lean mass, with replacement by fat tissue, have been reported in several studies on people with CP (15–17). It has been reported that children with CP present with greater intermuscular adiposity than the neurologically intact group (18). Adults with CP also show larger visceral and subcutaneous adiposity (4). Furthermore, the prevalence of sarcopenia in adults with CP is higher than that in the general population (19). Fat distribution may be particularly important in this population because of possible differences in body composition. Therefore, it is assumed that the regional fat distribution, as well as general body fat characteristics, may show a profile in people with CP that differs from that in the general population.

Therefore, we sought to identify the prevalence of obesity and the characteristics of body fat distribution in an adult population with CP, and we assessed their cardiovascular risks and the relationship thereof with body fat distribution in this population.

## Materials and Methods

### Participants

Participants were recruited from the community, with the cooperation of nationwide organizations for persons with disabilities, and four hospitals in Gyeonggi and Seoul in South Korea. A total of 243 adults with CP were included in this study. Participants were excluded if they were not able to understand or answer the questionnaire despite receiving assistance from an interviewer, if they failed to complete dual-energy X-ray absorptiometry (DXA), or if they withdrew before data collection. Data were collected between February 1, 2014, and November 31, 2014.

All study procedures were approved by the institutional review boards of the participating institutions, operating in compliance with the Guidelines for Good Clinical Practice. Written informed consent was obtained from all participants. After obtaining consent from the participants, questionnaire surveys on basic information, assessments, and measurements were conducted.

### Structured Interview and Physical Examination

A structured interview and physical examination were conducted by a physiatrist or a trained research nurse in order to complete the questionnaire regarding demographics and physical function. The questionnaire included questions on sex, age, current smoking status, and drinking habits. Current smoking was defined as any cigarette smoking within the previous month. Never cigarette smokers and ex-cigarette smokers were classified as non-smokers. Likewise, drinkers were classified as those with any alcohol consumption in the past previous month.

Waist circumference and resting blood pressure were also measured. Waist circumference was measured in subjects in a standing position, at normal expiration. It was measured at the midpoint between the lower margin of the least palpable rib and the top of the iliac crest, using a stretch-resistant tape (20), once for each participant. Systolic blood pressure (SBP; mmHg) was determined as the average of two measurements taken 1 min apart, with the subjects in the supine position, after subjects had rested quietly in a chair for at least 5 min. Treatment for hypertension was also recorded.

The types of CP and the areas affected were investigated. They were determined by a single physiatrist (SHJ) with more than 15 years of clinical experience in CP. The types of CP were classified as spastic, dystonic, dyskinetic, ataxic, or mixed (21). Affected areas were determined as quadriplegia, diplegia, hemiplegia, and monoplegia of the upper and lower extremities (22).

For gross motor function, we used the Gross Motor Function Classification System (GMFCS). This is a five-level scale, where level I represents the least disability and level V the most, based on typical performance rather than the maximal capacity (23, 24). People with GMFCS level I walk without limitations, whereas people with level V are transported in a manual wheelchair. It is widely used to describe abilities and limitations in gross motor function, including sitting and walking, in children and adolescents, aged up to 18 years, with CP (25). The subject's current and best previous GMFCS levels were determined by a physiatrist after a structured interview and clinical examination.

The age at deterioration of physical function was also examined. GMFCS levels in 10-year intervals were determined, and the age span of physical deterioration was defined as the period when there was a regression of GMFCS level. The participants were also categorized according to the GMFCS level: ambulatory (GMFCS levels I, II, and III) and non-ambulatory groups (GMFCS levels IV and V). History of fall and number of falls in the past year were recorded by interviewing the patients.

The Short Physical Performance Battery (SPPB) was assessed by a trained physiotherapist. It is a group of measures that combines the results of gait speed, chair stand, and balance tests (26). It is an important indicator of functional mobility and independence (26).

### Criteria for General Obesity

Basic body anthropometry was performed to measure height and body weight. Cutoffs for body mass index (BMI, kg/m^2^) were as follows: overweight (BMI of 25.0–29.9), and class 1, 2, and 3 obesity (BMI of 30–34.9, 35.0–39.9, and ≥40.0, respectively) (27). Fat mass index (FMI, kg/m^2^) was calculated as fat mass divided by the square of height. Cutoffs for FMI were as follows: fat deficit (<5%), normal (5%−9%), excess fat (>9%−13%), class I obesity (>13%−17%), and class II–III obesity (>17%) (28).

### Measurement of Body Fat Composition

**Figure 1:**
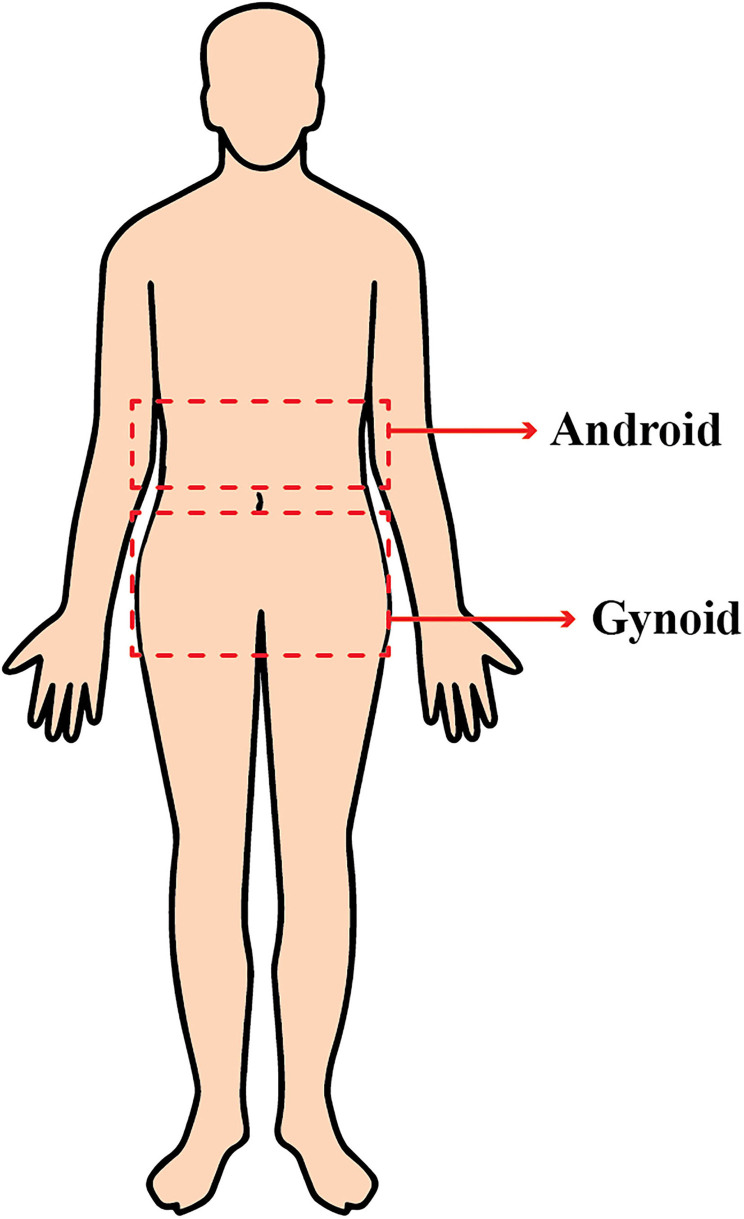
Android and gynoid body fat distribution.

For body composition assessment, DXA (GE Lunar Prodigy, Bedford, MA, United States) was used. DXA provides a precise evaluation of body composition at a relatively low cost (29). DXA differentiates bone mineral, lean, and fat soft tissues by measuring two different energy levels emitted from each type of tissue. The regions of interest (ROIs) were defined and calculated using the software provided by the manufacturer for local fat composition assessment. The android ROI was defined from the pelvis cut (lower boundary) to above the pelvis cut, by 20% of the distance. The gynoid area was from the lower boundary of the umbilicus ROI (upper boundary) to a line equal to two times the height of the android fat distribution ROI (lower boundary) (Figure 1).

### Laboratory Studies

A venous blood sample was obtained for laboratory analysis. The participants fasted for at least 8 hr before their blood was drawn. Blood composition analysis included total cholesterol and triglyceride (TG), high-density lipoprotein (HDL), low-density lipoprotein (LDL), and fasting plasma glucose (FPG) levels.

### Calculation of the Framingham Risk Score

The FRS has been widely used for the risk assessment of CVD (30, 31). The FRS was used to represent a participant's 10-year risk of coronary heart disease. The FRS was calculated using a web-based calculator (http://cvdrisk.nhlbi.nih.gov). This tool was designed for adults aged 20 years and older. The FRS estimates the 10-year coronary heart disease risk based on predictors, such as sex, age, total cholesterol, HDL, SBP, treatment for hypertension, and smoking status (32).

### Statistical Analysis

Means ± SD and 95% confidence intervals (CIs) were calculated for continuous variables. The clinical characteristics were compared between groups using an independent *t*-test for continuous variables and Student's *t*-test or Fisher's exact test for categorical variables. Adjustment of alpha level was not made for multiple comparisons in this study, as the authors assumed that it may lead to fewer errors in interpretation (33).

Associations between the FRS and other factors were examined using univariable and multiple regression analyses. To determine the factors independently associated with the FRS, variables with *p* <0.05 on univariable regression analysis were used for multiple regression analysis.

All *p*-values were calculated from two-tailed tests of statistical significance, with significance declared at the 5% level. All statistical analyses were conducted using the SPSS version 20.0 (SPSS Inc., Chicago, IL, United States).

## Results

### Characteristics of the Participants

Ninety-nine adults with CP were enrolled; however, 79 adults were included in the analysis in this study. DXA could not be performed in 20 adults. In 17 adults, precise measurement was not possible because of deformities and abnormal postures. Two adults had dystonic-type CP and one adult had athetoid-type CP and could not remain still during the measurement. The mean age of the study population (45 men and 34 women) was 42.2 ± 8.5 (95% CI, 40.3–44.2) years. The participant's characteristics, physical functions, and laboratory results are shown in Table 1.

**Table 1:** Participant's characteristics, physical function, and laboratory results.

	**Total (*N =* 79)**	**Men (*N =* 45)**	**Women (*N =* 34)**
**Age (year)**	42.2 ± 8.5 (40.3–44.2)	42.7 ± 9.9 (39.8–45.7)	41.6 ± 6.4 (39.3–43.8)
**Smoking** **(*N =* 77)**			
Current smoker	15 (19%)	14 (31%)	1 (3%)
**Alcohol** **(*N =* 74)**			
Drinker	43 (54%)	26 (61%)	17 (55%)
**Types of CP**			
Spastic	27 (34%)	16 (36%)	11 (32%)
Dystonic	13 (17%)	9 (20%)	4 (12%)
Dyskinetic	7 (9%)	3 (6%)	4 (12%)
Ataxic	1 (1%)	1 (2%)	0
Mixed	31 (39%)	16 (36%)	15 (44%)
**Affected area**		**(*****N** **=*** **42)**	**(*****N** **=*** **34)**
**(*****N** **=*** 76)^*^			
Quadriplegic	46 (60%)	23 (52%)	23 (72%)
Diplegic–both LE	12 (16%)	10 (23%)	2 (6%)
Diplegic–both UE	6 (8%)	2 (5%)	4 (13%)
Hemiplegic	8 (10%)	7 (16%)	1 (3%)
Monoplegic	4 (5%)	2 (4%)	2 (6%)
**Current GMFCS level**			
I	10 (13%)	4 (9%)	6 (18%)
II	21 (26%)	9 (20%)	12 (35%)
III	4 (5%)	4 (9%)	0
IV	41 (52%)	28 (62%)	13 (38%)
V	3 (4%)	0	3 (9%)
**Best previous GMFCS level**	**(*****N** **=*** 73)^*^	**(*****N** **=*** **42)**	**(*****N** **=*** **31)**
I	14 (19%)	8 (19%)	6 (19%)
II	30 (41%)	12 (29%)	18 (58%)
III	5 (7%)	5 (12%)	0
IV	22 (30%)	17 (40%)	5 (16%)
V	2 (3%)	0	2 (7%)
**Age at physical function deterioration**	**(*****N** **=*** 61)^*^	**(*****N** **=*** **32)**	**(*****N** **=*** **29)**
<10 (year)	7 (12%)	2 (6%)	5 (17%)
10–20 (year)	6 (10%)	3 (9%)	3 (10%)
20–30 (year)	16 (26%)	8 (25%)	8 (28%)
30–40 (year)	19 (31%)	11 (35%)	8 (28%)
40–50 (year)	10 (16%)	5 (16%)	5 (17%)
50 < (year)	3 (5%)	3 (9%)	0
**SPPB (*****N** **=*** **60)**	4.9 ± 4.7 (3.6–6.1)	4.0 ± 4.6 (2.4–5.6)	5.9 ± 4.7 (4.0–7.8)
**Number of falls in the past year**	1.5 ± 0.6 (1.3–1.6)	1.5 ± 0.6 (1.3–1.6)	1.4 ± 0.7 (1.2–1.7)
**Glucose (mg/dL)**	103.5 ± 21.3 (98.7–108.3)	104.9 ± 25.9 (97.1–112.7)	101.6 ± 13.3 (97.0–106.2)
**Albumin (g/dL)**	4.3 ± 0.2 (4.2–4.3)	4.4 ± 0.2 (4.3–4.4)	4.2 ± 0.2 (4.1–4.3)
**Triglycerides (mg/dL)**	136.6 ± 85.9 (117.4–155.9)	152.1 ± 90.5 (124.9–179.3)	116.1 ± 76.0 (89.6–142.7)
**Total cholesterol (mg/dL)**	175.4 ± 33.6 (167.9–182.9)	177.2 ± 35.0 (166.7–187.7)	173.1 ± 32.1 (161.9–184.3)
**HDL (mg/dL)**	48.4 ± 12.0 (45.7–51.1)	46.0 ± 9.3 (43.3–48.8)	51.5 ± 14.4 (46.5–56.6)
**LDL (mg/dL)**	99.1 ± 30.2 (92.3–105.8)	100.6 ± 28.4 (92.1–109.2)	97.0 ± 32.7 (85.6–108.4)

*CP, cerebral palsy; GMFCS, Gross Motor Function Classification System; SPPB, Short Physical Performance Battery; HDL, high-density lipoprotein; LDL, low-density lipoprotein*.

* *Missing data for each column are shown by the total number of participants*.

*Data are shown as mean ± SD (95% confidence intervals)*.

### Body Composition

There was no significant difference between sexes in waist circumference, BMI, BMI criteria, total body fat mass and fat percentage, and gynoid fat mass (Table 2). However, women had significantly higher gynoid fat mass, a higher percentage of gynoid and android body fat relative to the total body fat, and a lower android/gynoid fat ratio (*p* = 0.001, *p* = 0.003, *p* <0.001, respectively). The FMI was significantly higher in women than in men (*p* = 0.006). According to the FMI criteria, more men had a fat deficit than women (*p* = 0.002) (Table 2).

**Table 2:** Body anthropometry, body composition, Framingham risk score, and 10-year cardiovascular disease risk analysis by sex and ambulatory function.

	**Total (*N =* 79)**	**Men (*N =* 45)**	**Women (*N =* 34)**	***p*-value**	**Ambulatory group (*N =* 35)**	**Non-ambulatory group (*N =* 44)**	***p*-value**
**Waist circumference (cm)**	79.1 ± 18.8 (74.7–83.4)	77.3 ± 22.7 (70.3–84.4)	81.4 ± 12.0 (77.1–85.7)	0.363	82.8 ± 12.3 (78.3–87.4)	76.4 ± 22.1 (69.6–83.2)	0.145
**Body weight (kg)**	58.0 ± 12.8 (55.2–60.9)	61.2 ± 14.5 (56.9–65.6)	53.7 ± 8.5 (50.8–56.7)	0.009	59.2 ± 10.00 (55.8–62.6)	57.1 ± 14.6 (52.6–61.5)	0.467
**Body mass index (kg/m** ^2^ **)**	22.8 ± 4.6 (21.8–23.9)	23.0 ± 5.2 (21.4–24.5)	22.7 ± 3.6 (21.4–23.9)	0.774	23.2 ± 3.3 (22.0–24.3)	22.5 ± 5.4 (20.9–24.2)	0.523
Underweight (<18.5 kg/m^2^)	12(15%)	9(20%)	3(9%)	0.171	1(3%)	11(25%)	**0.006**
Normal (18.5–24.99 kg/m^2^)	45(57%)	22(49%)	23(67%)	0.095	24(69%)	21(47%)	0.063
Overweight (25–29.99 kg/m^2^)	16(20%)	10(22%)	6(18%)	0.616	8(23%)	8(18%)	0.608
Class I Obesity (30–34.99 kg/m^2^)	4(5%)	2(4.5%)	2(6%)	1	2(5%)	2(5%)	1
Class II Obesity (35–39.99 kg/m^2^)	2(3%)	2(4.5%)	0	0.503	0	2(5%)	0.5
Class III Obesity (≥ 40 kg/m^2^)	0	0	0	–	0	0	–
**Total body fat mass (kg)**	16.6 ± 8.9 (14.6–18.6)	15.0 ± 9.5 (12.1–17.9)	18.7 ± 7.8 (16.0–21.4)	0.071	18.0 ± 7.6 (15.4–20.6)	15.5 ± 9.9 (12.5–18.5)	0.211
**Android fat mass (g)**	1575.2 ± 973.2 (1357.2–1793.2)	1562.4 ± 1073.3 (12.4–18.8)	1592.1 ± 837.9 (1299.8–1884.5)	0.894	1690.2 ± 776.3 (1423.6–1956.9)	1483.7 ± 1105.5 (1147.6–1819.8)	0.352
**Gynoid fat mass (g)**	2979.5 ± 1430.5 (2659.1–3299.9)	2544.9 ± 1394.1 (2126.0–2963.7)	3554.7 ± 1283.2 (3107.0–4002.4)	**0.001**	3268.7 ± 1294.7 (2824.0–3713.5)	2749.4 ± 1504.7 (2292.0–3206.9)	0.109
**Percent body fat (%)**	27.6 ± 11.6 (25.0–30.2)	23.0 ± 10.3 (19.9–26.1)	33.6 ± 10.4 (30.0–37.3)	0.071	29.8 ± 9.6 (26.5–33.1)	25.9 ± 12.7 (21.9–29.8)	0.137
**Android body fat (% of total)**	9.4 ± 1.8 (9.0–9.8)	10.3 ± 1.5 (9.9–10.8)	8.3 ± 1.4 (7.8–8.8)	**<0.001**	9.5 ± 2.0 (8.8–10.2)	9.4 ± 1.6 (8.9–9.9)	0.798
**Gynoid body fat (% of total)**	18.7 ± 3.0 (18.0–19.3)	17.8 ± 2.7 (17.0–18.6)	19.8 ± 3.0 (18.7–20.8)	**0.003**	18.4 ± 2.7 (17.5–19.3)	18.9 ± 3.2 (17.9–19.8)	0.503
**Android/gynoid ratio**	0.5 ± 0.2 (0.5–0.6)	0.6 ± 0.2 (0.5–0.6)	0.4 ± 0.1 (0.4–0.5)	**<0.001**	0.5 ± 0.2 (0.5–0.6)	0.5 ± 0.2 (0.5–0.6)	0.683
**Fat mass index (kg/m** ^2^ **)**	6.6 ± 3.7 (5.8–7.4)	5.6 ± 3.6 (4.6–6.7)	7.9 ± 3.4 (6.7–9.1)	**0.006**	7.1 ± 3.1 (6.1–8.2)	6.2 ± 4.1 (5.0–7.4)	0.258
Fat deficit (<5)	27 (34%)	22 (49%)	5 (14%)	**0.002**	8 (23%)	19 (43%)	0.058
Normal (5–9)	37 (47%)	16 (35%)	21 (62%)	0.021	21 (60%)	16 (37%)	**0.036**
Excess fat (> 9 to 13)	8 (10%)	4 (9%)	4 (12%)	0.72	4 (11%)	4 (9%)	1
Class I Obesity (> 13 to 17)	6 (8%)	3 (7%)	3 (9%)	1	1 (3%)	5 (11%)	0.219
Class II–III Obesity (> 17)	1 (1%)	0	1 (3%)	0.43	1 (3%)	0	0.433
**Framingham risk score**	4.32 ± 5.22 (3.14–5.50)	5.20 ± 5.23 (3.63–6.77)	3.09 ± 5.02 (1.28–4.90)	0.081	4.00 ± 4.98 (2.34–5.66)	4.69 ± 5.81 (3.10–6.27)	0.84
**^†^10-year risk of developing CVD**	2.36 ± 4.01 (1.45–3.27)	3.88 ± 4.68 (2.48–5.29)	0.22 ± 0.49 (0.04–0.40)	**<0.001**	1.65 ± 2.85 (0.69–2.59)	3.09 ± 4.52 (1.86–4.33)	0.199

*CVD, Cardiovascular disease*.

† *Two patients were not included in the analysis as smoking history was not recorded*.

*Data are shown as the mean ± SD or percentage. The 95% confidence intervals (CI) around the mean are also shown*.

*Independent t-test was performed independently of sexes and ambulatory function*.

There was no significant difference between the ambulatory and non-ambulatory groups in waist circumference, BMI, and body fat composition. However, there was a higher proportion of underweight individuals, by BMI criteria, in the non-ambulatory group (*p* = 0.006) and a higher proportion of individuals with normal FMI in the ambulatory group (*p* = 0.036).

The 10-year risk of developing coronary heart disease was higher in men than in women (*p* <0.001). The FRS and 10-year risk of developing coronary heart disease did not differ between the ambulatory and non-ambulatory groups.

### Framingham Risk Score and Related Factors

Univariable regression analysis showed that FRS was positively associated with increasing age (*p* <0.001; Table 3). The FRS also increased as the percentage of android fat increased (*p* = 0.007). In women, the android/gynoid fat ratio was positively associated with FRS (*p* = 0.047).

**Table 3:** Univariable regression analyses for the Framingham risk score.

**Domain**	**Variable**	**Total**			**Men**			**Women**		
		**β**	**SE**	***p*-value**	**β**	**SE**	***p*-value**	**β**	**SE**	***p*-value**
Anthropometry	Age	0.429	−13.897	**<0.001**	0.348	−9.688	**<0.001**	0.693	−26.047	**<0.001**
	Body weight	−0.038	6.526	0.423	−0.092	10.813	0.093	0.033	1.36	0.783
	Waist circumference	−0.007	4.706	0.845	−0.001	5.368	0.977	0.036	−0.315	0.676
Physical function	GMFCS level	0.096	3.911	0.849	0.155	4.698	0.841	−0.284	3.679	0.676
	SPPB total score^†^	−0.077	4.418	0.614	−0.027	5.471	0.897	0.062	1.808	0.784
	Number of falls in the past year	−0.92	5.581	0.352	−0.715	6.293	0.607	−1.353	4.846	0.325
	Age at physical deterioration^‡^	1.122	0.58	0.027	1.336	0.82	0.065	0.555	1.274	0.442
Body fat amount and distribution parameters	Body mass index	−0.107	6.751	0.426	−0.234	10.565	0.122	0.296	−3.519	0.301
	Total fat mass	0	5.941	0.144	0	7.103	0.126	2.709	2.595	0.823
	Android fat mass	−0.01	5.145	0.397	−0.001	6.985	0.121	0.001	1.591	0.385
	Gynoid fat mass	−0.01	6.565	0.08	−0.001	7.232	0.161	0	3.458	0.896
	Android fat percent	0.897	−4.19	**0.007**	0.298	2.123	0.573	1.674	−10.835	**0.006**
	Gynoid fat percent	−0.269	9.334	0.184	0.057	4.177	0.848	−0.43	11.567	0.149
	Android/ gynoid fat ratio	6.563	0.829	0.066	0.158	5.106	0.975	12.803	−2.552	**0.047**
	Percent body fat	−0.067	6.217	0.201	−0.074	7.01	0.349	0.027	2.182	0.756
	Fat mass index	−0.222	5.773	0.181	−0.323	7.022	0.143	0.135	2.047	0.627

*GMFCS, Gross Motor Function Classification System; SPPB, Short Physical Performance*.

† *SPPB was not performed in 20 patients*.

‡ *There were 12 missing data*.

Data are shown as mean ± SD.

*β refers to the regression coefficient, and SE refers to the standard error of estimate*.

Multiple regression analysis of the FRS was performed with the factors (age and android fat percentage) that were significantly associated with FRS in univariable regression analysis. Multiple regression analysis showed that the FRS was associated with age and android fat percentage based on the formula below (*p* <0.001). R^2^ shows the percentage of variance in the outcome explained by all variables in the model.


$$\begin{array}{l} FRS\;=\;-18.549\;+\;0.410*\;Age\;+\;0.577\;*\;Android\;percent\;fat\\ \;\left(\%\right)\;(R^{2}\;=\;0.528)\; \end{array}$$

## Discussion

This study shows that age and android fat percentage are independently associated with CVD risk in adults with CP. On the other hand, factors such as BMI, GMFCS level, and functional abilities were not found to be related to CVD risk in adults with CP. Notably, the CVD risk was significantly associated with the android fat proportion rather than the measures of overall adiposity, such as BMI and total body fat, in adults with CP.

Age and disproportionate distribution of body fat were the major predictors of CVD risk in this study. It is widely accepted that the risk of CVD increases with age (34–36). The American Heart Association (AHA) reports that the incidence of CVD is ca. 40% from 40 to 59 years, ca. 75% from 60 to 79 years, and ca. 86% in those older than 80 years (37). Recently, disproportionate fat distribution has been suggested as an important factor predicting CVD risk (38, 39). Although the underlying mechanism of the associations between regional adiposity and CVD risk is not yet clear, regional body fat distribution around the abdominal area is known to be related to metabolic syndromes, such as dyslipidemia, hypertension, and type 2 diabetes mellitus (40) even in normal-weight people, children, and older individuals (11, 41, 42). It has been reported that android body fat is strongly associated with circulating levels of CRP and fibrinogen, thus increasing the risk of subclinical inflammation, leading to endothelial dysfunction (41).

In this study, body fat distribution was different between sexes, while BMI and total body fat did not differ. Women showed a markedly higher 10-year risk of CVD than men. These results are in line with those of the general population. Fat distribution differs between sexes in non-abled populations (43, 44). CVD is markedly more common in men in the general population (45). The reasons for the sex differences have not yet been fully elucidated (46). However, it has been suggested that android fat distribution may contribute to metabolic disturbances that affect CVD risk (47, 48). One of the suggested reasons for regional fat differences is sex hormones (49). Female sex hormones are known to cause the accumulation of body fat in the lower body regions, which is essential for reproductive function (50, 51). This may account for one of the reasons for the difference in CVD risk between the sexes (36).

Overweight and obesity rates based on general obesity measures were 22% in the study population. According to the Organization for Economic Co-operation and Development (OECD) reports released in 2012, the average overweight and obesity rate in South Korea was 35.1%. We found that Korean adults with CP in this study were not obese compared to the general Korean population. It has been debated whether adults with CP are more obese than the general population. Most studies have reported that adults with CP are more likely to be obese due to a lack of physical activity and a sedentary lifestyle (2, 5, 52–54). As we focused on individuals who were able to participate in the survey, those with intellectual disabilities were not included, and this may account for the different results, as obesity rate in adults with CP is known to be closely related to intellectual disability (54). Previous studies did not exclude those with intellectual disabilities (2, 5, 52–54). Studies by van der Slot et al. showed that the obesity rate is slightly lower in adults with CP than in the general population (2). In the study by Van der Slot, the included subjects were relatively young, with ages ranging from to 25 to 45 years, and those with severe intellectual disabilities were excluded. In addition, since most of the previous studies investigating obesity among patients with CP have been conducted in Western countries, the results of our study on the Korean population could be different due to cultural differences or eating behaviors. Likewise, in a study on the growth profile assessment of adults with tethered cord syndrome in Korea, these subjects had lower height, weight, and BMI than controls of the same age (55), which differ from the previous results of higher rate of obesity among spinal bifida patients in Western countries (56, 57). It is conceivable that since the participants in this study had relatively diverse CP types and function levels, the risk of undernutrition due to dysphagia or feeding problems also existed.

On the other hand, the FRS scores in this population group were higher than those in the general Korean population. Park et al. (58) showed that according to the 2012 Korea National Health and Nutrition Examination Survey, the FRS in the general population was 2.85, while that of our study population was 4.4, which was higher than that of the general population. These results were in line with previous reports that the CVD risk is higher in adults with CP (1, 3, 5). It should be noted that the overall obesity rate was lower in adults with CP than in the general population. This further indicates that general obesity may not be very predictive of CVD risk in adults with CP.

The discrepancy between BMI and FRS may underestimate the risk of metabolic disease in adults with CP who have normal or low BMI. The reason for the discordance may be explained by the body fat distribution, because age and android fat percentage were the factors that were associated with FRS, while factors such as BMI, GMFCS, or functional abilities were not found to be related to FRS.

Due to altered body morphology and changes in lifestyle over a long period, the measures of overall adiposity, such as BMI, would not be appropriate for adults with CP. In adults with CP, higher excess adiposity can be detected despite a normal BMI when compared to neurologically intact adults. Therefore, it is important to evaluate fat distribution rather than general adiposity measures in this population. Peterson et al. assessed intermuscular adipose tissue and trunk adiposity using abdominal computed tomography (CT) (4). CT can distinguish between visceral adipose tissue and subcutaneous adipose tissue, while DXA can assess body compartment compositions, such as the android and gynoid areas. There are several limitations to the use of CT scans for body composition assessment. There could be a potential concern for over- or underestimation of fat tissue, as only the selected levels of fat area were measured. In addition, CT has greater radiation hazards than DXA (59). With DXA, bone density or skeletal muscle mass can also be measured, as osteoporosis and sarcopenia are other conditions that should be considered in adults with CP (5).

In our study, it is notable that the ambulatory group had a higher proportion of individuals with a normal FMI. Moreover, both the fat-deficit and obese groups (according to FMI) were higher in the non-ambulatory group. On the other hand, the CVD risk by FRS was not significantly different between the non-ambulatory and ambulatory groups. The non-ambulatory group may be at risk of potential undernutrition caused by dysphagia and gastrointestinal problems, resulting in a fat deficit (6, 7). On the other hand, decreased levels of physical activity may lead to excess fat deposits. The ambulatory group may have led a lifestyle similar to the general population and were likely to be more “fit” than the non-ambulatory group. However, there was no significant difference in CVD risk according to the ambulatory status in this study. Heyn et al. showed that those with GMFCS level III had an increased CVD risk when compared to those with GMFCS levels I and II. The average age of the study group in Heyn et al. study was 24 ± 5 years. In contrast, the average age was relatively older in our study (42.2 ± 8.5 years). The age of the study group may have affected the general activity level, even if they had the same GMFCS level. In addition, the relatively small number of participants may have been insufficient to show a statistically significant difference.

This study had some limitations. First, this was a cross-sectional study. Therefore, we were unable to determine a cause-and-effect relationship. Second, a head-to-head comparison with the general population was not performed. Instead, we were only able to compare our data with the general population data from the OECD reports. Since the cohort represents a sample that is relatively small for determining prevalence in the CP population, a future study with a larger sample size would address this limitation. Third, other information, such as socioeconomic status or nutritional status, was not analyzed in this study. With these considerations, further detailed studies on body fat distribution and CVD risk in adults with CP could be performed.

In conclusion, android body fat distribution and age are the two significant factors associated with 10-year CVD risk in adults with CP. Body fat distribution is more closely related to CVD risk than measures of general obesity in adults with CP.

## Data Availability Statement

The raw data supporting the conclusions of this article will be made available by the authors, without undue reservation.

## Ethics Statement

The studies involving human participants were reviewed and approved by the Institutional Review Board at Seoul National University Boramae Medical Center. All subjects provided their written informed consent to participate in this study.

## Author Contributions

SJ and HS: drafted the manuscript. SJ: conceived the study and critically revised the manuscript for important intellectual content. All authors have read and approved the manuscript and agreed to be accountable for all aspects of the work in ensuring that questions related to the accuracy or integrity of any part of the work are appropriately investigated and resolved.

## Funding

This research was supported by the Translational R&D Program on Smart Rehabilitation Exercises (#TRSRE-PS01), National Rehabilitation Center, Ministry of Health & Welfare, Korea.

## Conflict of Interest

The authors declare that the research was conducted in the absence of any commercial or financial relationships that could be construed as a potential conflict of interest.

## Publisher's Note

All claims expressed in this article are solely those of the authors and do not necessarily represent those of their affiliated organizations, or those of the publisher, the editors and the reviewers. Any product that may be evaluated in this article, or claim that may be made by its manufacturer, is not guaranteed or endorsed by the publisher.
